# 

**Published:** 1874-04

**Authors:** 


### ACKNOWLEDGMENTS.

Atlanta Herald, Daily; Rome Courier, Daily; Philadelphia Medical Times; The Clinic; Medical & Surgical Reporter; Wilson's Herald of Health; Nashville Journal of Medicine and Surgery; Chicago Medical Journal; Medical Examiner; Detroit Review of Medicine and Pharmacy; Illustrated Annual of Phrenology; Baltimore Physician and Surgeon; Boston Journal of Chemistry; The Medical Record; American Journal of Pharmacy; Druggist's Circular; St. Louis Medical and Surgical Reporter; Boston Medical and Surgical Journal; The Druggist; Cincinnati Lancet and Observer; Medical News and Library; American Practitioner; University Record, Sewanee; North Western Medical and Surgical Journal; New Orleans Medical and Surgical Journal; Physician and Pharmacist; Popular Science Monthly; Indiana Journal of Medicine; Buffalo Medical and Surgical Journal; London Lancet; Western Lancet.

